# Urinary symmetric dimethylarginine: A promising biomarker for acute kidney injury in dogs with pyometra

**DOI:** 10.1002/vetr.70733

**Published:** 2026-05-19

**Authors:** Larissa Ayane do Nascimento Braz, Suellen Rodrigues Maia, Lara Vilela Soares, Natani Silva Reis, Leandro Zuccolotto Crivellenti, Andrigo Barboza de Nardi

**Affiliations:** ^1^ Department of Clinical and Veterinary Surgery, School of Agricultural and Veterinary Sciences Universidade Estadual Paulista ‘Júlio de Mesquita Filho’ Jaboticabal Brazil; ^2^ Department of Veterinary Medicine Federal Institute of Southern Minas Gerais Muzambinho Brazil; ^3^ Graduate Program in Veterinary Science, College of Veterinary Medicine Universidade Federal de Uberlândia Uberlândia Brazil

**Keywords:** acute kidney injury, biomarkers, canine, histopathology, nephrology

## Abstract

**Background:**

Symmetric dimethylarginine (SDMA) is a biomarker of renal function derived from arginine methylation, unaffected by muscle mass or diet, and increases after only a 25% reduction in renal function, allowing earlier detection of renal dysfunction compared with serum creatinine. Although serum SDMA is well established in veterinary nephrology, urinary SDMA (uSDMA) remains comparatively underexplored, particularly in dogs with acute kidney injury (AKI). This study evaluated uSDMA concentrations and the uSDMA‐to‐creatinine (uSDMA/Cr) ratio as biomarkers for AKI, using renal histopathology as the reference standard.

**Methods:**

Twenty‐seven non‐azotemic dogs diagnosed with pyometra were evaluated. uSDMA was measured using ELISA, and renal lesions were histopathologically classified as discrete, moderate or severe.

**Results:**

Median uSDMA concentrations were significantly higher in dogs with severe lesions (0.43 µg/mL, interquartile range [IQR] 0.40–0.47) compared with those with moderate lesions (0.31 µg/mL, IQR 0.24–0.38) and discrete lesions (0.26 µg/mL, IQR 0.22–0.30) (*p* < 0.001). The uSDMA/Cr ratio was lower in the discrete group (0.0031, IQR 0.0026–0.0036) than in the moderate group (0.0039, IQR 0.0032–0.0048) and the severe group (0.0069, IQR 0.0058–0.0080) (*p* = 0.0198).

**Limitations:**

The main limitations include the absence of a healthy control group and lack of serial biomarker measurements, which may restrict the assessment of dynamic changes.

**Conclusions:**

These preliminary findings suggest that uSDMA and uSDMA/Cr ratio may serve as biomarkers for assessing AKI severity in dogs, supporting earlier detection and monitoring.

## INTRODUCTION

Acute kidney injury (AKI) is one of the most challenging conditions in veterinary clinical practice due to its high mortality, non‐specific clinical signs and diagnostic difficulties, especially in early grades.^1^, ^2^ Currently, the diagnosis and staging of AKI in dogs rely on serum creatinine levels—a late biomarker influenced by extrarenal factors such as muscle mass, hydration status and nutritional condition, which limit diagnostic accuracy.^3^, ^4^, ^5^ These limitations highlight the need for earlier, more sensitive, biomarkers to detect kidney dysfunction at early stages.

Serum symmetric dimethylarginine (SDMA) is a small, methylated arginine derivative (∼202 Da), released during intranuclear protein turnover and eliminated almost exclusively by the kidneys. SDMA is freely filtered by the glomerulus and, under normal physiological conditions, is neither reabsorbed nor actively secreted by the renal tubules. These characteristics make SDMA a reliable surrogate marker of glomerular filtration rate. Unlike creatinine, SDMA is minimally affected by muscle mass, diet or body condition and increases after an estimated 25% reduction in renal function, demonstrating greater sensitivity for early detection of kidney dysfunction.^6^, ^7^ Although serum SDMA has been widely investigated in chronic kidney disease (CKD) and is increasingly evaluated in AKI studies,^2^, ^8^ current evidence focuses predominantly on serum concentrations.

To our knowledge, urinary SDMA (uSDMA) has not previously been evaluated in dogs. Although the mechanisms regulating urinary elimination of SDMA under pathological conditions remain unclear, urinary excretion may be altered in the presence of renal injury, reflecting changes in renal handling rather than glomerular filtration alone. Therefore, uSDMA may provide complementary information to serum SDMA, particularly in non‐azotemic patients. Normalisation to urinary creatinine concentration (uSDMA/Cr) may further reduce variability related to urine dilution and improve biomarker interpretability.^9^

Pyometra is a common reproductive disorder in intact female dogs, frequently associated with systemic inflammation and renal injury. It represents a clinically relevant model for evaluating kidney damage under naturally occurring infectious conditions.^10^, ^11^ Therefore, this study aimed to assess the association between uSDMA (absolute and normalised) and histopathological kidney lesion severity in non‐azotemic dogs diagnosed with pyometra.

Histopathological evaluation enables direct assessment of lesion severity and represents the reference standard for diagnosing renal damage.^10^, ^12^ We hypothesised that uSDMA concentrations and the uSDMA‐to‐creatinine ratio (uSDMA/Cr) would be higher in dogs presenting more severe renal histopathological lesions despite normal serum creatinine.

## METHODS

### Study design

This prospective cross‐sectional observational study included non‐azotemic female dogs diagnosed with pyometra. Dogs were admitted for surgical management (ovariohysterectomy) and were included if they met the following criteria: (1) serum creatinine concentration 1.6 mg/dL or less; (2) confirmed diagnosis of pyometra based on clinical evaluation, ultrasonographic evidence of uterine enlargement with intrauterine purulent content, and positive bacterial culture of uterine fluid; and (3) absence of clinical, laboratory or ultrasonographic evidence of CKD. Animals with chronic illnesses, endocrinopathies, neoplasia or other relevant comorbidities were excluded.

At admission, all dogs underwent a complete clinical evaluation, including measurement of heart rate, respiratory rate, rectal temperature and systolic arterial blood pressure. Age, breed and bodyweight were also recorded. Due to variability in owner‐reported history at presentation, the exact duration of clinical signs prior to admission could not be reliably determined.

Owner‐informed consent was obtained before enrollment, and the institutional animal ethics committee approved all procedures.

### Laboratory procedures

Blood samples were collected preoperatively, before anaesthesia and any treatment. Urinalysis was performed on samples obtained by cystocentesis during ovariohysterectomy. Preoperative cystocentesis was avoided due to the risk of uterine perforation in advanced pyometra.

#### Haematological analysis

Complete blood counts were performed using an automated cell counter (PocH‐100iv Diff), while leukocyte differentials were manually analysed using Rosenfeld‐stained blood smears.

#### Biochemical analysis

Serum biochemical parameters, including creatinine, urea, SDMA, phosphorus, total calcium, total protein (g/dL), albumin (g/dL) and globulins (g/dL), were analysed to assess renal function using an automated spectrophotometer (LabQuest, LABTEST). Serum SDMA concentrations were measured by a species‐specific immunoassay kit (Bioassay Technology Laboratory; sensitivity 2.45 nmol/L; range 5–1500 nmol/L; intra‐/inter‐assay coefficients of variation (CVs) <8% and <10%; validation data as reported by the manufacturer).

#### Urinalysis

Urinalysis included physical, chemical and microscopic evaluations of fresh urine samples obtained by cystocentesis during ovariohysterectomy. Urine samples were centrifuged, and the supernatants were stored in liquid nitrogen (–196°C) for up to 12 months without freeze–thaw cycles before SDMA analysis.

#### Urinary SDMA measurement

uSDMA was assessed using a canine‐specific ELISA kit (Bioassay Technology Laboratory; sensitivity 2.45 nmol/L; range 5–1500 nmol/L; intra‐/inter‐assay CVs <8% and <10%; validation data as reported by the manufacturer). The samples were run in duplicate.

### Renal biopsy and histopathological assessments

Before biopsy, all dogs underwent preoperative safety screening to minimise procedural risk. This evaluation included a complete physical examination (rectal temperature, heart and respiratory rates), platelet count and systolic arterial blood pressure measurement performed in triplicate using Doppler ultrasonography. Primary haemostasis was assessed using platelet count and buccal mucosal bleeding time. Dogs presenting abnormalities that increased anaesthetic or surgical risk were excluded from biopsy.

Renal biopsies were obtained during ovariohysterectomy performed for the treatment of pyometra, allowing controlled collection of renal cortical tissue from the left kidney using a Tru‐Cut biopsy needle (14G or 16G, depending on the patient's size), thereby avoiding an additional anaesthetic procedure. After renal biopsy, all animals were monitored at 24, 48 and 72 hours, and periodically for up to 15 days. Follow‐up evaluations included physical examination, packed cell volume measurement and abdominal ultrasonography to detect any potential post‐procedural complications.

Kidney samples fixed in Bouin's solution were sectioned (2–3 µm) and stained with haematoxylin and eosin, periodic acid‐Schiff, Jones methenamine silver, Masson's trichrome and Congo red (10 µm). At least two samples containing more than 10 glomeruli were analysed by light microscopy. Two blinded nephropathologists examined paraffin sections, according to the standards of the International Veterinary Renal Pathology Service (IVRPS).^13^ Evaluated lesions included tubular injury (TI), interstitial fibrosis and tubular atrophy (IFTA), interstitial (INFInf) and periglomerular (InfPG) inflammatory infiltrates, membranoproliferative glomerulonephritis (MPGN) and glomerular sclerosis.

TI was graded from 0 (none) to 3 (severe) based on features such as epithelial cell swelling, apical blebs, brush border loss, basement membrane irregularities, necrosis and presence of tubular casts. IFTA was defined by decreased tubular density, atrophy and expansion of the interstitium by fibrous tissue. INFInf was characterised by infiltration of mononuclear cells (mainly lymphocytes and plasma cells) into the interstitial space, whereas InfPG showed similar infiltrates concentrated around glomeruli. Both were scored semi‐quantitatively (absent, discrete, moderate, diffuse) or by estimating the percentage of affected tissue, and categorised into a 0–3 scale (0: ≤5%, 1: 6%–25%, 2: 26%–50%, 3: >50%). MPGN was identified by mesangial cell proliferation, matrix expansion and thickening of the glomerular basement membrane, often accompanied by partial or complete obliteration of capillary lumina. Glomerular sclerosis was defined by global hyalinisation of the tuft and absence of visible capillary spaces. InfPG, MPGN and glomerular sclerosis were all graded according to the proportion of glomeruli affected, using the same 0–3 scale.

### Experimental groups

Female dogs diagnosed with pyometra were classified according to the severity of renal histopathological changes using a cumulative lesion classification system developed and applied by the authors for the present study, based on semi‐quantitative lesion scores assigned in accordance with the guidelines of IVRPS.^13^ This scoring method used the total number of renal microscopic lesions to stratify into three groups: discrete (D, total score ≤2), moderate (M, total score 3–4) and severe (S, total score ≥5).

### Statistical analysis

All the data were analysed in R (version 4.1.1). Normality was assessed using the Shapiro–Wilk test, and as most variables did not meet normal distribution assumptions, non‐parametric tests were applied. Group comparisons were performed using the Kruskal–Wallis test followed by Dunn's post hoc test. Variables are presented as median (interquartile range [IQR]), and statistical significance was defined as a *p*‐value of 0.05 or less.

We did not perform a formal sample size calculation, as all cases were derived from routine clinical presentations of pyometra. However, a post hoc power analysis based on uSDMA values (group medians, IQR, and sample sizes of 8, 8 and 11) yielded a high effect size (*f* = 8.55) and a power of 1.0, indicating that the sample size was sufficient to detect statistically significant differences for this variable.

## RESULTS

### Clinical and epidemiological parameters

The age of the dogs ranged from 3 to 18 years, with no significant differences between groups (*p* > 0.05). Most dogs were mixed breed (11/27), with the purebred dogs, including Maltese, Yorkshire Terrier, Fila Brasileiro, English Bulldog, Pekingese, Pug, Golden Retriever, Poodle, Pinscher and Labrador Retriever. Bodyweight did not differ significantly among groups, with median values of 15.5 kg (IQR 9.58–23.13) in the discrete group, 7.35 kg (IQR 4.55–27.28) in the moderate group, and 8.40 kg (IQR 5.85–17.50) in the severe group (*p* = 0.628). No significant intergroup differences were observed for heart rate, respiratory rate, blood pressure or body temperature (all *p* > 0.05).

### Histopathological findings and lesion classification

Histopathological analysis revealed tubular lesions in 85% (23/27) of pyometra cases, which were classified as discrete (48%), moderate (35%) or severe (17%). The lesions were characterised by epithelial degeneration, vacuolisation, denudation and focal necrosis (Figure 1). IFTAs were observed in 48% of the samples, predominantly those with discrete (54%) or moderate (33%) lesions. Inflammatory infiltrates (67%) were mainly mononuclear, with different degrees of interstitial oedema. Glomerular changes included MPGN (63%) and sclerosis (37%), predominantly classified as discrete lesions.

**FIGURE 1:**
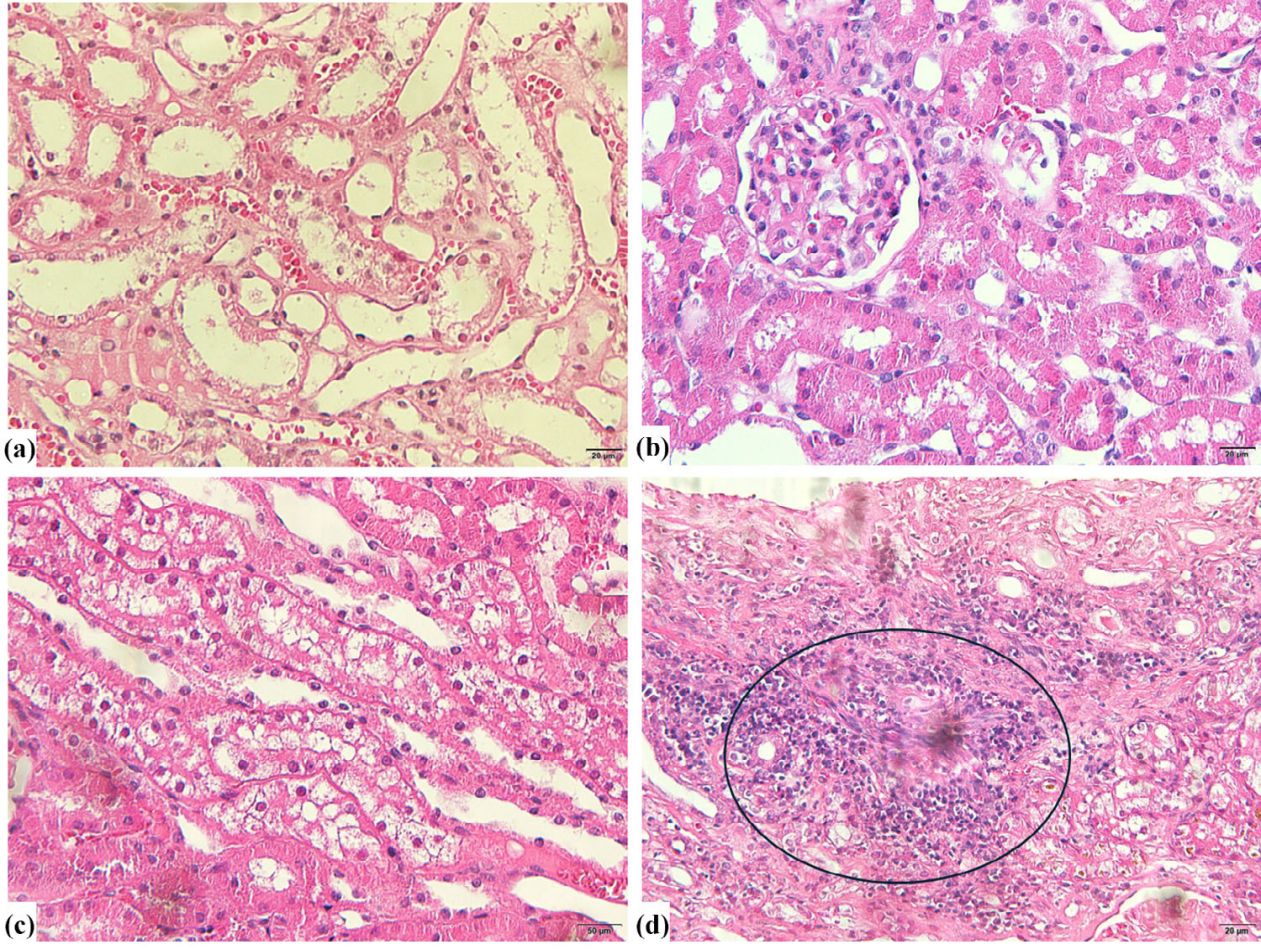
Representative photomicrographs of renal cortical tissue illustrating the grading scale for tubular injury (TI) and interstitial inflammatory infiltrate (INFInf). (A) TI grade 0. Normal tubular morphology, with preserved epithelial lining and open tubular lumina, indicating absence of TI. Haematoxylin and eosin (H&E) stain, 400× magnification; bar = 50 µm. (B) TI grade 1. Mild TI characterised by focal tubular dilatation, slight epithelial cell flattening and discrete cellular swelling. Tubular architecture mostly preserved. H&E stain, 400× magnification; bar = 50 µm. (C) TI grade 2. Moderate TI with hydropic degeneration, marked cytoplasmic vacuolisation, loss of brush border and reduction of tubular luminal space. H&E stain, 400× magnification; bar = 50 µm. (D) TI grade 3 and INFInf grade 3. Severe tubulointerstitial injury characterised by diffuse mononuclear inflammatory infiltrate, tubular epithelial degeneration, atrophy and collapse of tubular structures. H&E stain, 400× magnification; bar = 20 µm.

Dogs were classified into experimental groups according to histopathological severity: discrete (*n* = 8), moderate (*n* = 8) and severe (*n* = 11). No biopsy‐related complications were observed during the 15‐day postoperative monitoring period. Individual lesion scores are provided in Table S1.

### Blood counts

Blood counts did not differ significantly between the discrete, moderate and severe groups (Table 1; *p* > 0.05).

**TABLE 1:** Median (interquartile range) of laboratory parameters in dogs diagnosed with pyometra, grouped according to renal histopathological severity (discrete ≤2, moderate 3–4, severe ≥5).

Parameter	Reference interval	Discrete	Moderate	Severe	*p*‐Value
Blood count					
Red blood cells (×10^6^/µL)	5.5–8.5	5.66 (3.74–6.44)	5.75 (4.93–6.94)	6.30 (5.25–6.75)	0.5819
Haemoglobin (g/dL)	12–18	13.50 (8.30–14.35)	12.25 (11.08–16.75)	13.40 (7.80–15.05)	0.7756
Haematocrit (%)	37–55	38.15 (14.65–41.55)	36.10 (33.58–47.20)	39.40 (32.45–41.80)	0.5873
MCV (fL)	60–77	65.90 (64.44–71.00)	66.25 (63.63–68.58)	62.20 (61.35–65.50)	0.1453
MCHC (g/dL)	32–36	34.19 (33.75–34.74)	35.30 (34.60–35.90)	35.50 (34.30–36.25)	0.3793
Platelets (×10^3^/µL)	200–500	279.50 (256.50–481.25)	342.00 (161.50–416.00)	240.00 (189.00–376.00)	0.5227
Leukocytes (×10^3^/µL)	6–17	24.35 (21.75–41.20)	24.45 (15.98–45.90)	27.40 (14.60–49.60)	0.9470
Eosinophils (×10^3^/µL)	0–1.5	0.80 (0.21–1.00)	0.09 (0.00–0.49)	0.00 (0.00–0.29)	0.0890
Band neutrophils (×10^3^/µL)	0–0.3	0.21 (0.00–0.60)	1.29 (0.36–2.20)	0.81 (0.49–2.51)	0.1779
Segmented neutrophils (×10^3^/µL)	3–11.5	18.17 (15.98–24.44)	19.76 (11.55–37.85)	22.74 (10.62–38.86)	0.9807
Lymphocytes (×10^3^/µL)	1–4.8	2.66 (2.03–3.57)	2.73 (2.32–3.25)	4.03 (2.47–7.11)	0.4313
Monocytes (×10^3^/µL)	0.2–1.4	0.30 (0.00–0.87)	0.11 (0.00–0.49)	0.14 (0.00–1.11)	0.7420
Metamyelocytes (×10^3^/µL)	0	0.00 (0.00–0.00)	0.00 (0.00–0.00)	0.00 (0.00–0.00)	0.9530
Biochemical analysis					
Creatinine (mg/dL)	0.5–1.4	0.91 (0.76–1.11)	0.80 (0.63–1.00)	0.90 (0.70–1.00)	0.9283
Urea (mg/dL)	10–60	20.25 (16.10–25.25)^b^	24.00 (17.75–29.50)^a^ ^b^	36.00 (29.60–57.50)^a^	0.0358
Total protein (g/dL)	5.5–7.5	7.75 (6.90–8.10)	7.50 (6.20–9.07)	7.50 (6.75–8.10)	0.6871
Albumin (g/dL)	2.5–4.0	2.27 (1.95–2.65)	2.30 (2.05–2.57)	2.40 (2.00–2.79)	0.4094
Globulins (g/dL)	2.0–4.0	5.83 (4.57–7.03)	4.56 (3.60–6.35)	4.60 (3.85–6.14)	0.4724
Total calcium (mg/dL)	9.0–11.5	9.20 (8.35–11.70)	9.20 (8.55–10.17)	10.70 (8.80–13.15)	0.5230
Phosphorus (mg/dL)	2.5–6.0	6.30 (4.75–7.85)	5.10 (4.75–5.62)	5.75 (3.75–11.87)	0.7380
Lactate (mmol/L)	0.5–2.5	1.88 (1.83–1.94)	2.11 (1.70–2.52)	2.01 (1.50–3.01)	0.7019
Urinalysis					
UPC (ratio)	<0.5	0.13 (0.00–0.43)	0.18 (0.05–0.35)	0.50 (0.24–0.75)	0.1119
Urine‐specific gravity	>1.030	1.028 (1.026–1.030)^a^	1.028 (1.015–1.042)^a^	1.012 (1.008–1.016)^b^	0.0022
Urinary creatinine^*^ (mg/dL)	50–200	85.2 (69.2–101.3)	78.5 (56.5–100.5)	62.0 (48.0–76.1)	0.041
SDMA analysis					
Serum SDMA (µg/mL)	0.5–1.2	0.24 (0.22–0.25)	0.17 (0.14–0.25)	0.23 (0.19–0.27)	0.1819
Urinary SDMA (µg/mL)	–	0.26 (0.22–0.30)^b^	0.31 (0.24–0.38)^b^	0.43 (0.40–0.47)^a^	0.0006
uSDMA/Cr ratio	–	0.0031 (0.0026–0.0036)^b^	0.0039 (0.0032–0.0048)^b^	0.0069 (0.0058–0.0080)^a^	0.0001

*Note*: Urinary reference intervals may vary based on hydration status and renal concentrating ability. Different superscript letters (a and b) within the same row indicate statistically significant differences according to the Kruskal–Wallis test followed by Dunn's post hoc test. Used to normalize urinary biomarker concentrations and to calculate the uSDMA/Cr ratio; not interpreted as an isolated diagnostic variable.

Abbreviations: Cr, creatinine; MCHC, mean corpuscular haemoglobin concentration; MCV, mean corpuscular volume; SDMA, symmetric dimethylarginine; UPC, urine protein‐to‐creatinine ratio; uSDMA, urinary symmetric dimethylarginine.

^*^ Indicates that urinary creatinine was used to normalize urinary biomarker concentrations and to calculate the uSDMA/Cr ratio. Statistically significant differences are indicated by different superscript letters (a and b) within the same row.

### Biochemical and urinalysis

Serum biochemical parameters were similar between groups (*p* > 0.05), except for urea, which was higher in the severe group (median 36.00 mg/dL, IQR 29.60–57.50) than in the discrete group (median 20.25 mg/dL, IQR 16.10–25.25) (*p* = 0.0358). Urinalysis revealed a significant difference in urine‐specific gravity (*p* = 0.0022), with lower values in the severe group (Table 1).

### SDMA analysis

The median uSDMA levels were significantly higher in the severe group (0.43 µg/mL, IQR 0.40–0.47) than in the discrete group (0.26 µg/mL, IQR 0.22–0.30) and the moderate group (0.31 µg/mL, IQR 0.24–0.38) (*p* = 0.0006). The median uSDMA/creatinine ratio was lower in the discrete group (0.0031, IQR 0.0026–0.0036) than in the moderate group (0.0039, IQR 0.0032–0.0048) and the severe group (0.0069, IQR 0.0058–0.0080) (*p* = 0.0198). Biplot analysis demonstrated that uSDMA and the uSDMA/creatinine ratio distinguished renal lesion severity (Figure 2).

**FIGURE 2:**
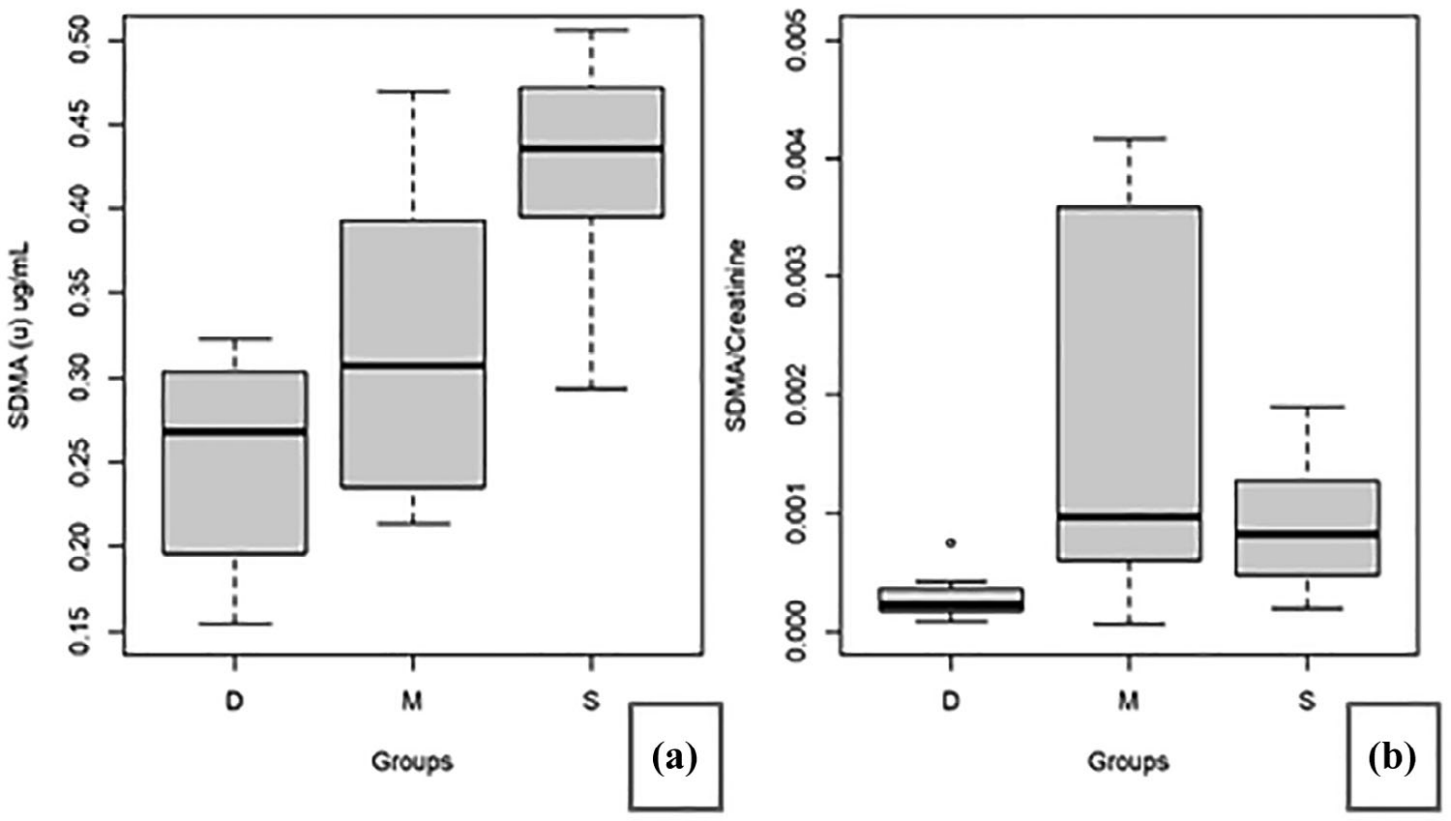
Boxplots of (A) urinary symmetric dimethylarginine (uSDMA) concentration and (B) uSDMA‐to‐creatinine ratio in dogs with pyometra, grouped according to histopathological severity (D: discrete, M: moderate, S: severe).

## DISCUSSION

This study demonstrated that uSDMA concentrations and the uSDMA‐to‐creatinine ratio were significantly higher in dogs with more severe renal histopathological lesions, despite all animals being non‐azotemic at admission. In contrast, serum SDMA and serum creatinine levels did not differentiate between lesion severity groups. These results suggest that uSDMA may serve as a potential biomarker of renal injury, particularly when functional markers remain within normal limits.

Pyometra was selected as a clinically relevant model for investigating early renal injury as its association with renal complications, including AKI, is well established.^10^, ^11^ Additionally, surgical treatment of pyometra enables ethical and practical access to renal tissue without additional procedures.^10^ The study population consisted of routine clinical cases that reflect real‐world diagnostic challenges.

Histopathological evaluation confirmed that all dogs had renal lesions of varying severity, underscoring the importance of early biomarker detection even in non‐azotemic patients.

Although mild chronic changes, such as focal interstitial fibrosis or mild glomerulosclerosis, were identified in some animals, these findings are frequently described in clinically healthy adult dogs and were not predominant.^14^ None of the dogs showed clinical, ultrasonographic or laboratory evidence of CKD at admission, making pre‐existing CKD an unlikely contributor to the observed renal alterations. The main histopathological findings included tubular epithelial degeneration, cellular debris, luminal casts, interstitial oedema and inflammatory infiltrates, which are characteristic of acute or recent injury rather than chronic degenerative processes.^12^

Although some authors have reported promising results regarding the use of serum SDMA in AKI assessment,^15^ evidence remains inconsistent, particularly when compared with histopathological findings, which are considered the reference standard for determining renal injury severity. In the present study, serum SDMA did not differ significantly between groups, consistent with previous studies indicating that SDMA is predominantly a marker of glomerular filtration rate and may lack sensitivity to detect early‐grade AKI.^2^, ^8^ This supports the concept that serum biomarkers primarily reflect renal function through glomerular filtration, whereas urinary biomarkers may provide complementary information on structural injury and TI, potentially increasing sensitivity for detecting early or subclinical damage.^4^, ^5^, ^7^

In contrast, uSDMA differentiated between lesion severity groups. The concentrations of uSDMA were higher in dogs with severe histopathological lesions, suggesting that elevated uSDMA may indicate structural renal injury before changes in serum biomarkers become apparent. The uSDMA/Cr ratio further improved discrimination between groups by adjusting for urine dilution effects.^9^

When establishing urinary biomarkers for the identification of AKI, it is important to understand the mechanisms by which urinary biomarker concentrations change during disease. For example, urinary cystatin C is elevated due to impaired tubular reabsorption of this low molecular weight protein.^4^ During periods of reduced glomerular filtration and tubular damage, one might expect lower urinary concentrations of SDMA, given that it is freely filtered by the glomerulus and is neither reabsorbed nor actively secreted by the renal tubules.^5^ Thus, the finding of elevated uSDMA concentrations during AKI suggests that alternative mechanisms may be involved. One possible explanation is that structural damage to renal tubular epithelial cells leads to local release or altered renal excretion of SDMA. Alternatively, local inflammation during renal injury may contribute to increased SDMA release.

Despite tubular and glomerular lesions, serum creatinine concentrations remained within normal limits across all groups, supporting the concept that serum creatinine is a late marker of renal dysfunction and may not reflect early or subclinical injury. This highlights the diagnostic value of urinary biomarkers as indicators of renal structural damage in non‐azotemic dogs.

An important strength of this study is the inclusion of non‐azotemic dogs and the application of renal histopathology as the reference standard. Histopathological assessment is rarely performed in clinical investigations but allows direct evaluation of renal structural injury. This approach enabled the identification of structural damage before functional impairment, strengthening the evidence that urinary biomarkers, such as uSDMA, may facilitate early detection of clinically silent renal injury and support timely intervention.

However, some limitations should be considered. The absence of a control group without pyometra limited the interpretability of comparisons, although such a population would not accurately reflect the diagnostic challenges of AKI in clinical settings. Serial biomarker evaluations were not performed, which limited the assessment of biomarker dynamics over time. In addition, although urine culture was conducted at admission, these data were not included as urinary infection was not a primary focus of the study. Therefore, the potential influence of concurrent or subclinical urinary tract infection on urinary biomarker concentrations cannot be entirely excluded and should be addressed in future investigations. Additionally, although inflammatory processes associated with pyometra may contribute to alterations in urinary biomarker levels, this interaction was not assessed and requires further investigation. Longitudinal studies incorporating multiple urinary biomarkers across different clinical models are needed to better characterise the diagnostic performance of uSDMA.

In conclusion, uSDMA concentrations and the uSDMA/creatinine ratio were significantly associated with the severity of renal histopathological lesions in non‐azotemic dogs with pyometra. The ability to detect structural renal damage in the absence of azotaemia highlights the potential of uSDMA as an indicator of early renal injury, warranting further investigation across broader clinical contexts.

## AUTHOR CONTRIBUTIONS

*Concept and design of the study, funding acquisition, supervision, data interpretation, revision and final approval of the manuscript*: Leandro Zuccolotto Crivellenti. *Conceptualisation, data collection, formal analysis, software management, visualisation, original draft preparation and manuscript review*: Larissa Ayane do Nascimento Braz. *Data curation, methodology and investigation*: Suellen Rodrigues Maia. *Visualisation and manuscript review and editing*: Lara Vilela Soares. *Manuscript review and editing*: Natani Silva Reis. *Scientific supervision and manuscript review and editing*: Andrigo Barboza de Nardi.

## CONFLICT OF INTEREST STATEMENT

The authors declare they have no conflicts of interest.

## FUNDING INFORMATION

This study was supported by the Syão Paulo Research Foundation (FAPESP), Brazil (grant no. 17/25458‐0), and by the Minas Gerais State Research Foundation (FAPEMIG), in partnership with the National Council for Scientific and Technological Development (CNPq), Brazil (grant no. APQ‐06621‐24). Larissa Ayane do Nascimento Braz received a doctoral scholarship from the Coordination for the Improvement of Higher Education Personnel (CAPES), Brazil (Finance Code 88887.610798/2021‐00).

## ETHICS STATEMENT

The current study protocol was approved by the Institutional Animal Care and Use Committee of the Faculty of Agricultural and Veterinary Sciences, Universidade Estadual Paulista/Jaboticabal, Brazil (approval numbers: 1855/21 and 4979280318).

## Supporting information




**Supporting File 1**: vetr70733‐sup‐0001‐SuppMat.docx

## Data Availability

The data that support the findings of this study are available from the corresponding author upon reasonable request.
