# Supplementary material for: Urinary symmetric dimethylarginine: A promising biomarker for acute kidney injury in dogs with pyometra
Source: Vet Rec. 2026 May 19;199(7):e337–44. doi: 10.1002/vetr.70733 (PMC13631850; doi:10.1002/vetr.70733)
Supplement: Supplementary file 1 [file VETR-199--s001.docx]

**Supplementary Table 1.** Histopathological data results from the experiment on dogs with pyometra were scored according to the severity of each lesion, in accordance with the "International Veterinary Renal Pathology Service" (IVRPS). Lesions were classified as ≤ 2 (D: discrete), 3–5 (M: moderate), and ≥5 (S: severe)

| Patient | INJTub | FIAT | INFInf | InfPGl | MPGN | Esc | Sum | Group |
| --- | --- | --- | --- | --- | --- | --- | --- | --- |
| 1 | 1 | 0 | 1 | 0 | 0 | 0 | 2 | D |
| 2 | 2 | 1 | 0 | 0 | 0 | 0 | 3 | M |
| 3 | 2 | 2 | 0 | 0 | 2 | 0 | 6 | S |
| 4 | 1 | 0 | 1 | 0 | 0 | 0 | 2 | D |
| 5 | 1 | 0 | 1 | 0 | 2 | 0 | 4 | M |
| 6 | 0 | 0 | 0 | 1 | 0 | 1 | 2 | D |
| 7 | 3 | 2 | 2 | 0 | 3 | 3 | 13 | S |
| 8 | 2 | 0 | 0 | 0 | 3 | 1 | 6 | S |
| 9 | 1 | 1 | 0 | 1 | 0 | 0 | 3 | M |
| 10 | 2 | 2 | 2 | 2 | 2 | 1 | 11 | S |
| 11 | 2 | 2 | 2 | 1 | 1 | 1 | 9 | S |
| 12 | 2 | 0 | 1 | 0 | 0 | 2 | 4 | M |
| 13 | 1 | 0 | 1 | 1 | 1 | 0 | 4 | M |
| 14 | 1 | 1 | 0 | 0 | 1 | 1 | 4 | M |
| 15 | 2 | 2 | 2 | 0 | 2 | 0 | 8 | S |
| 16 | 1 | 0 | 1 | 1 | 1 | 0 | 4 | M |
| 17 | 2 | 0 | 1 | 1 | 2 | 1 | 7 | S |
| 18 | 3 | 2 | 3 | 3 | 3 | 0 | 14 | S |
| 19 | 3 | 1 | 1 | 1 | 2 | 0 | 8 | S |
| 20 | 1 | 0 | 0 | 1 | 0 | 0 | 2 | D |
| 21 | 3 | 1 | 2 | 2 | 3 | 0 | 11 | S |
| 22 | 1 | 0 | 1 | 1 | 3 | 1 | 7 | S |
| 23 | 1 | 1 | 0 | 1 | 0 | 1 | 4 | M |
| 24 | 1 | 0 | 1 | 0 | 0 | 0 | 2 | D |
| 25 | 0 | 0 | 0 | 0 | 1 | 0 | 1 | D |
| 26 | 0 | 1 | 1 | 0 | 1 | 0 | 3 | D |
| 27 | 0 | 0 | 1 | 1 | 0 | 0 | 2 | D |

INJTub, Tubular injury; FIAT, Fibrosis and atrophy; INFInf, Interstitial inflammatory infiltrates, graded based on the extent of inflammatory cell infiltration in the interstitium; InfPGl, Periglomerular infiltrates; MPGN, Membranoproliferative glomerulonephritis; Esc, Glomerular sclerosis. Som, Summation. D: discrete, M: moderate, S: severe.
